# A gossypol biosynthetic intermediate disturbs plant defence response

**DOI:** 10.1098/rstb.2018.0319

**Published:** 2019-01-14

**Authors:** Xiu Tian, Xin Fang, Jin-Quan Huang, Ling-Jian Wang, Ying-Bo Mao, Xiao-Ya Chen

**Affiliations:** 1 School of Life Sciences, Nanjing University, Nanjing 210023, People's Republic of China; 2 National Key Laboratory of Plant Molecular Genetics, Chinese Academy of Sciences, Shanghai 200032, People's Republic of China; 3 CAS Key Laboratory of Insect Developmental and Evolutionary Biology, CAS Center for Excellence in Molecular Plant Sciences, Shanghai Institute of Plant Physiology and Ecology, Chinese Academy of Sciences, Shanghai 200032, People's Republic of China

**Keywords:** secondary metabolism, disease resistance, Arabidopsis, cotton, gossypol, reactive electrophile species

## Abstract

Plant secondary metabolites and their biosynthesis have attracted great interest, but investigations of the activities of hidden intermediates remain rare. Gossypol and related sesquiterpenes are the major phytoalexins in cotton. Among the six biosynthetic intermediates recently identified, 8-hydroxy-7-keto-δ-cadinene (C234) crippled the plant disease resistance when accumulated upon gene silencing. C234 harbours an α,β-unsaturated carbonyl thus is a reactive electrophile species. Here, we show that C234 application also dampened the *Arabidopsis* resistance against the bacterial pathogen *Pseudomonas syringae* pv. *maculicola* (*Psm*). We treated *Arabidopsis* with C234, *Psm* and (*Psm*+C234), and analysed the leaf transcriptomes. While C234 alone exerted a mild effect, it greatly stimulated an over-response to the pathogen. Of the 7335 genes affected in the (*Psm*+C234)-treated leaves, 3476 were unresponsive without the chemical, in which such functional categories as ‘nucleotides transport’, ‘vesicle transport’, ‘MAP kinases’, ‘G-proteins’, ‘protein assembly and cofactor ligation’ and ‘light reaction’ were enriched, suggesting that C234 disturbed certain physiological processes and the protein complex assembly, leading to distorted defence response and decreased disease resistance. As C234 is efficiently metabolized by CYP71BE79, plants of cotton lineage have evolved a highly active enzyme to prevent the phytotoxic intermediate accumulation during gossypol pathway evolution.

This article is part of the theme issue ‘Biotic signalling sheds light on smart pest management’.

## 1. Introduction

As sessile organisms, plants protect themselves from herbivores and pathogens by synthesizing structurally diversified secondary (specialized) metabolites, many of which exert defence function by their cytotoxicity [1]. However, these compounds could be harmful to host cells too, and plants have evolved sophisticated mechanisms to avoid self-toxicity of these metabolites [2]. It is well acknowledged that the toxic metabolites can be accumulated and stored in specific structures, such as the glandular trichome, laticifer, or transformed into a non-toxic form by modification such as glycosylation, representing physical and chemical strategies the plant has developed to overcome self-toxicity [3].

The biosynthesis of defence compounds in plants mostly involves intermediates, which may also have biological activities. There is growing evidence to indicate that over-accumulation of intermediates in plants could result in disturbance of plant growth and development [4,5]. However, until now elucidation of the molecular basis of the activities of toxic intermediates has been rare.

Sesquiterpenoids, biosynthesized from the 15-carbon farnesyl diphosphate (FDP), constitute one of the largest families of natural products of plants, many of which function as signalling molecules in bio-interactions or as defence compounds (phytoalexins) to safeguard the plants [1]. Some sesquiterpenes are volatile, others are stored in plants as derivatives decorated by subsequent reactions. The sesquiterpenoid phytoalexins, such as gossypol, capsidiol and zealexin in cotton, tobacco and maize (*Zea mays*) plants, respectively, have been investigated in depth [6–8]. Cotton species belong to the genus *Gossypium*, family Malvaceae. In cotton plants gossypol and related sesquiterpene aldehydes are the major phytoalexins against pathogens and pests. They are generally toxic [9,10] and stored in pigmented glands of aerial organs and in epidermal layers of roots [6].

**Figure 1.**
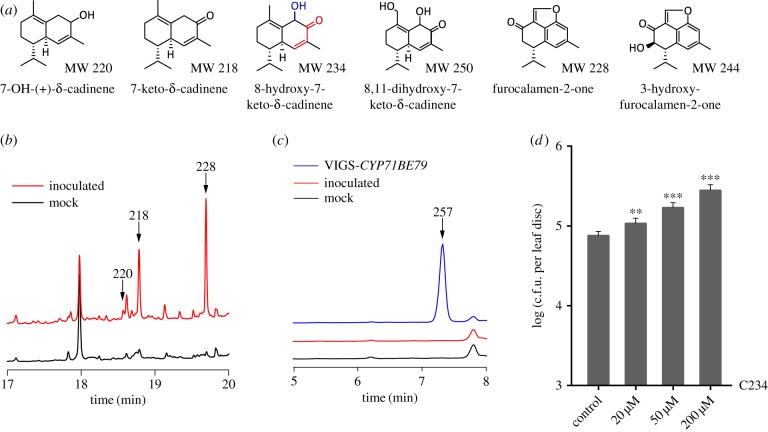
The gossypol biosynthetic intermediate 8-hydroxy-7-keto-δ-cadinene (C234) affects plant disease resistance. (*a*) Structures of six gossypol biosynthetic intermediates. The α,β-unsaturated carbonyl group in C234 is indicated in red; (*b*) GC-MS of the respective metabolites in the hypocotyl-root junction of the control and the *R. solani*-inoculated plants. The intermediates are represented with the *m/z* (mass-to-charge ratio) values shown in (*a*); (*c*) LC-MS of the metabolites in the hypocotyl-root junction of the control and the plants inoculated with *R. solani*: 8-hydroxy-7-keto-δ-cadinene was detected after *CYP71BE79* silencing (VIGS-*CYP71BE79*), but not in control and *R. solani*-inoculated samples; (*d*) pathogen growth. Leaves of the four-week-old *Arabidopsis* were inoculated with *Psm* ES4326 (OD_600_ = 0.0001). The *in planta* bacterial titres were determined at 3 dpi colony forming units (c.f.u.). Data represent the mean of six independent samples with standard deviation (**p < 0.02, ***p < 0.001, Student's *t* test). Experiments were repeated three times with similar results.

The biosynthesis of gossypol starts with the conversion of FDP into (+)-δ-cadinene, catalysed by the sesquiterpene cyclase (+)-δ-cadinene synthase [11,12]. We recently reported the characterization of five hydroxylation/oxidation steps that modify the (+)-δ-cadinene skeleton [13]. After virus-induced gene silencing (VIGS) of the corresponding enzymes, six intermediates were isolated [13]. One of them, 8-hydroxy-7-keto-δ-cadinene (C234), bears an α,β-unsaturated carbonyl adjacent to a hydroxyl group (figure 1*a*). Compounds containing the α,β-unsaturated carbonyl or other reactive electrophilic atom groups are classified as reactive electrophile species (RES) [14]. When the *CYP71BE79* expression was repressed by VIGS, C234 accumulated and the plant developed brown sunken lesions covering the hypocotyl-root junction [13]. Furthermore, the enzyme CYP71BE79 exhibited an exceptionally high activity in C234 hydroxylation and is evolutionally more conserved than other enzymes of the gossypol pathway [13]. Therefore, C234 has interesting biological activities worthy of further investigation.

In this research, we used *Arabidopsis thaliana* to explore the biologic activities of C234 in plant defence. *Pseudomonas syringae* pv. *maculicola* (*Psm*) ES4326 is a bacterial pathogen of cruciferous plants, including *A. thaliana*. We examined the plant responses to C234, *Psm* and both (*Psm*+C234). The global changes of the *Arabidopsis* transcriptomes analysed by RNA-sequencing (RNA-seq) provide a broader view of the interplay between C234 and plant immunity, and the possible mechanism of interfering disease resistance by the RES metabolite.

## 2. Material and methods

### (a) Plant materials and growth conditions

Plants of upland cotton, *Gossypium hirsutum* cv. R15, were grown in a greenhouse at 28 ± 2°C under a 14 h light photoperiod, and plants of *A. thaliana* (ecotype Col-0) were grown at 22°C and 16 h light photoperiod.

### (b) Pathogen infection and plant treatments

*Rhizoctonia solani* was cultured on potato dextrose agar medium at 28°C for 48 h. The 3-week old cotton plants grown in sterilized soil were inoculated with *R. solani* as described [15], and analysed at indicated days post inoculation (dpi).

The 4-week old plants of *A. thaliana* were infected with *Psm* ES4326 (OD_600_ = 0.0001), as described [16]. The compound C234 (8-hydroxy-7-keto-δ-cadinene), dissolved in dimethyl sulfoxide (DSMO), was added to buffer (10 mM MgSO_4_) or the bacterial solution to a final concentration of 20, 50 and 200 µM, respectively, before infiltration into two rosette leaves (leaf numbers 5–6). The same exogenous application of DMSO in buffer served as mock (control treatment). For each independent experiment, at least 20 replicate leaves from 10 plants per treatment were measured before performing a statistical analysis. All pathogen experiments depicted in the figures were repeated several times with similar results.

### (c) RNA isolation, RNA-sequencing and transcriptome analysis

Total RNAs were isolated using the RNAprep Pure Plant Kit (Tiangen) from the treated leaf tissues of the mock and the treated plants with three biological replicates for each treatment, according to the manufacturer's instructions. Library construction and sequencing were carried out using NEBNext^®^ Ultra™ RNA Library Prep Kit for Illumina^®^ (NEB, USA). The mRNA was prepared by using oligo (dT) magnetic beads and interrupted into short fragments (125 bp) in the fragmentation buffer. The first-strand cDNA was synthesized by random hexamers (mRNA fragments as templates). After second-strand cDNA synthesis and adaptor ligation, the cDNA fragments of 150–200 bp were isolated with AMPure XP system (Beckman Coulter, Beverly, USA), followed by purification and polymerase chain reaction (PCR)-enrichment to create the final cDNA library. After quality checking by an Agilent 2100 Bioanalyzer, the samples were sequenced on a Hiseq X Ten platform (Illumina) at Novogene Bioinformatics Institute, Beijing, China.

For each sample, we obtained approximately 50 million raw reads, which were processed through in-house perl scripts to remove the adapter sequences, reads containing ploy-N and low-quality bases to generate the clean data (clean reads). Using the tool of bowtie/tophat2 (http://tophat.cbcb.umd.edu/), about 93% of the useful reads could be uniquely mapped to the *A. thaliana* TAIR10.20 coding sequence. Gene annotation was referred to databases of Ensembl (http://www.ensembl.org/), KEGG (http://www.genome.jp/kegg/), and eggnog (http://eggnog.embl.de/). Gene expressions were normalized and calculated as readcount values for each gene with the DESeq package [17]. The significantly differentially expressed genes (fold change > 1.5 or < 0.67; *p* adjusted value < 0.05) were selected by pairwise comparison, clustered by Cluster 3.0 with Pearson distance and pairwise centred-linkage as clustering or hierarchical clustering methods, and viewed by TreeView. The *Arabidopsis* transcripts were annotated with descriptions from TAIR10 and functional annotations from MapMan. To determine the proportions of the C234 and *Psm* responsive genes in gene families (http://www.arabidopsis.org/), MapMan categories and the respective gene sets were aligned to the RNA-seq datasets using Microsoft Excel [18].

### (d) Quantitative reverse transcriptase PCR

The cDNAs were synthesized from 2 µg RNAs by genomic DNA removal and cDNA synthesis kit (Transgene, Beijing), followed by amplification with gene-specific primers designed according to National Center for Biotechnology Information (NCBI) Primer-Blast (www.ncbi.nlm.nih.gov/tools/primer-blast/). The quantitative reverse transcriptase PCR (qRT-PCR) was performed on a Bio-Rad CFX Connect Real-Time PCR system (Bio-Rad, USA) using SYBR green PCR Mix (TAKARA), according to the manufacturer's instructions for standard two-step amplification programme. *Arabidopsis thaliana UBIQUITIN 5* (*UBQ5*) was used as an internal reference. Primers used in this investigation are listed (see the electronic supplementary material, table S1).

### (e) Analysis of metabolites

Fresh plant tissues, 0.1 g, were ground in liquid nitrogen, extracted with 1.5 ml hexane containing 2 ng µl^−1^ nonyl acetate as an internal standard with shaking at 25 Hz for 30 min. Extracts were analysed by gas chromatography-mass spectrometry (GC-MS) on an Agilent 6890 Series GC System coupled to an Agilent 5973 Network Mass Selective Detector, using the following programme: initial temperature 60°C (5 min hold), increase to 180°C at 10°C min^−1^, to 240°C at 20°C min^−1^, and ramp to 280°C at 30°C min^−1^ (5 min hold). The flow rate of the carriage gas (He) was 1 ml min^−1^. Split injection (split ratio 5 : 2). The MS data between *m/z* 30-550 were recorded.

For liquid chromatography mass spectrometry (LC-MS) analysis, samples were extracted with 1 ml methanol and analysed by reversed-phase LC on an Agilent 1200 high performance liquid chromatography, using a Thermo Syncronis C18 analytical column (150 × 4.6 mm, 5 µm). Water with 0.1% formic acid (A) and acetonitrile with 0.1% FA (B) (positive ion mode) were used as the mobile phase components at a flow rate of 1 ml min^−1^ with the following 10 min gradient: 0–3 min, 20–70% B; 3–5 min, 70–80% B; 5–7 min, 80–84% B; 7–8 min, 84–100% B; 8–10 min, 100–20% B. A coupled Agilent 6120 Quadrupole LC/MS spectrometer collected the MS data in positive ion mode (parameters: mass range: 100–1000 *m/z*; drying gas: 350°C, 12 l min^−1^; nebuliser: 50 psig; capillary: 5000 V; fragmentor: 70 V). Each run of the first 2 min was discarded to avoid contamination of the apparatus.

### (f) Accession numbers

The sequencing data have been deposited in the NCBI (https://www.ncbi.nlm.nih.gov/) under the accession numbers of SRR7686004–SRR7686015.

## 3. Results

### (a) The gossypol biosynthetic intermediate C234 reduces plant disease resistance

As reported previously, cotton plants showed enhanced susceptibility to the soil-borne necrotrophic fungus *R. solani* following *CYP71BE79*-sliencing, which caused the accumulation of the substrate C234 (8-hydroxy-7-keto-δ-cadinene), particularly in the hypocotyl and root (electronic supplementary material, figure S1). Biosynthesis of gossypol in cotton is markedly induced upon pathogen infection or elicitor treatments [19]. To examine the effect of *R. solani* infection on the accumulation of gossypol biosynthetic intermediates, we compared the metabolites of leaf extracts from the *R. solani*-inoculated and the control (buffer-treated) cotton plants. GC-MS and LC-MS analyses showed that most of the intermediates were undetectable in the hypocotyl-root junction of the cotton plants; however, after inoculation with *R. solani*, several metabolites became detectable and their levels elevated (figure 1*b*). A notable exception was C234, which remained undetectable after the pathogen inoculation (figure 1*c*). These results demonstrated that the C234 was detectable only after the *CYP71BE79* gene silencing. As silencing other genes of gossypol biosynthesis did not promote the *R. solani* infection and symptom development [13], the accumulation of C234 should be responsible for reduced disease resistance of the cotton plant. We hypothesized that the metabolites induced to accumulate might have little or positive effect on plant defence against *R. solani*, whereas C234 affected disease resistance negatively.

To test whether the influence of C234 on plant defence is general or specific to cotton, we examined its activity on *Arabidopsis*. We found that treatment with C234 rendered the *A. thaliana* plants significantly more susceptible to the bacterial pathogen *Psm* ES4326, and the susceptibility increased with the C234 concentrations ranging from 20 to 200 µM (figure 1*d*). On the other hand, when added to culture medium it had no obvious effect on bacterial growth in our culture conditions (electronic supplementary material, figure S2), indicating that this keto-bearing compound does not inhibit the bacterial growth directly. Together, these data suggest that the gossypol pathway intermediate C234 dampens plant disease resistance in a way that appears general and independent of the gossypol biosynthesis.

### (b) C234 overstimulates the transcriptome changes during plant defence

**Figure 2.**
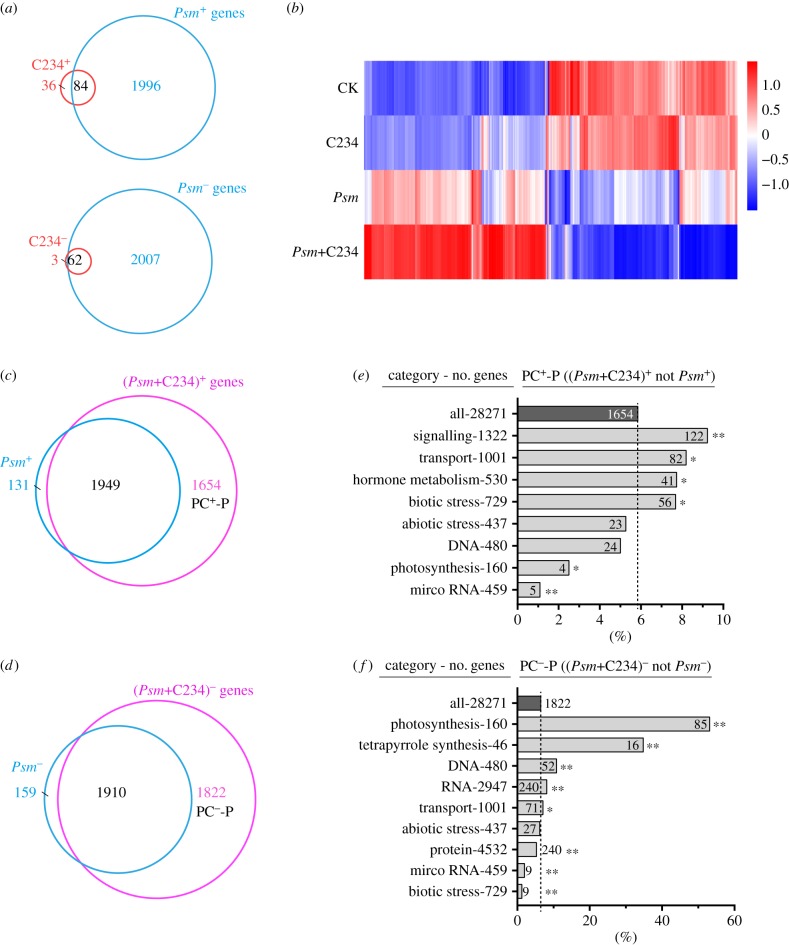
The transcriptional responses of *Arabidopsis* leaves to C234, *Psm* and (*Psm*+C234). (*a*) Venn diagrams indicating overlaps between C234^+^ and *Psm*^+^ (up) and C234^−^ and *Psm*^−^ genes (down). Red: C234-regulated genes, blue: *Psm*-regulated genes. (*b*) Heatmap of differentially expressed genes in the mock (CK), C234-, *Psm*- and (*Psm*+C234)-treated leaves. (*c*,*d*) Venn diagrams indicating overlaps between the upregulated *Psm*^+^, (*Psm*+C234)^+^ and (*Psm*+C234)^+^-*Psm* (PC^+^-P) genes (*c*), and the downregulated *Psm*^−^, (*Psm*+C234)^−^ and (*Psm*+C234)^−^-*Psm* (PC^−^-P) genes (*d*). Blue: *Psm*-responsive genes, magenta: (*Psm*+C234)-responsive genes. (*e*,*f*) Proportions of PC^+^-P (*e*) and PC^−^-P (*f*) genes in main MapMan functional categories. The total number (no.) of genes in each category is indicated on the left. The absolute number of PC^+^-P and PC^−^-P genes within a particular category is indicated on the horizontal bars. Asterisks indicate significant enrichment (or depletion) of gene categories in PC^+^-P or PC^−^-P genes (*p < 0.05; **p < 0.001; Fisher's exact test).

To further assess the impacts of C234 on plant defence, we treated the *Arabidopsis* plants with C234 infiltration and *Psm* inoculation, and analysed the responses in leaves two days later by RNA-seq. We directly compared the transcriptional changes after C234 treatment and *Psm*-inoculation, i.e. the response of leaves towards a localized inoculation (figure 2*a*). Compared to the control, there were 120 genes upregulated and 65 genes downregulated in the C234-treated samples, much less than the genes affected by *Psm*-infection (2080 upregulated and 2069 downregulated). This drastic difference suggests a narrower physiological response of the plant to the compound C234 than to the pathogen *Psm*.

To examine the consequence of C234 treatment on plant defence response to the pathogen, we added C234 to the *Psm* solution and analysed the transcriptional changes of the plants in response to (*Psm*+C234). Compared to the control, there is a total of 7335 genes differentially expressed in the (*Psm*+C234)-treated samples (3603 (*Psm*+C234)^+^ and 3732 (*Psm*+C234)^−^), much more than the genes affected by the *Psm* inoculation alone (figure 2*b–d*). By Venn diagram, we compared (*Psm*+C234)^+^ with *Psm*^+^, (*Psm*+C234)^−^ with *Psm*^−^, respectively. Of the 3603 (*Psm*+C234)^+^ genes, 1654 responded to (*Psm*+C234) but not to *Psm* (PC^+^-P), and, on the other side, 1822 of the (*Psm*+C234)^−^ genes were not repressed by *Psm* (PC^−^-P). The significant increase in gene numbers of PC^+^/^−^-P than those of *Psm*^+^/^−^ suggested that the compound C234 greatly broadened the plant response to the pathogen. We then focused on the differentially regulated genes, particularly those extended by C234, to gain insights into the functionality of C234 in plant defence.

### (c) C234 affects multiple physiological processes in a pathogen-challenged plant

We next examined the C234 responsive genes significantly enriched or depleted in particular MapMan categories and *Arabidopsis* gene families (http://www.arabidopsis.org/). Among the main MapMan bins, the categories of ‘biotic stress’ and ‘abiotic stress’ showed the greatest enrichment among the C234^+^ genes (electronic supplementary material, figure S3). These terms were also enriched among the *Psm*^+^ genes (electronic supplementary material, figure S3). A number of the C234-inducible genes have been shown to act in plant defence, including, for instance, genes involved in the biosynthesis of floral homoterpene volatiles (terpene synthase 04 (*TPS04*) and *CYP82G1*) [20,21] and the phytoalexin camalexin (*CYP71B15*) [22,23], and in disease resistance such as pathogenesis-related *PR1* [24]. In our RNA-seq data these genes were also responsive to *Psm* and showed stronger response in the (*Psm*+C234)-inoculated plants (electronic supplementary material, table S2). These results are consistent with the previous observations that the α,β-unsaturated carbonyl-containing compounds are highly active and potent stimulators of the pathogenesis-related genes [25,26].

**Figure 3.**
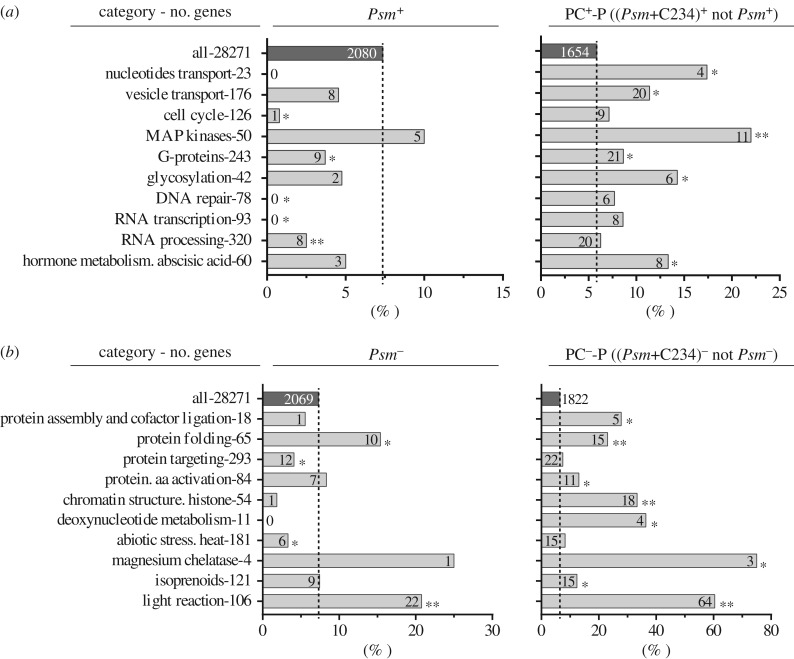
Main MapMan functional categories. Proportions of genes of PC^+^-P (*a*) and PC^−^-P (*b*) compared with those of *Psm*^+^ (*a*) and *Psm*^−^ (*b*), respectively. Dashed vertical lines illustrate the percentage of PC^+^-P (PC^−^-P) and *Psm*^+^ (*Psm*^−^) genes in the whole of the RNA-seq-covered transcriptome (28271 genes). The total number (no.) of genes in each category is indicated on the left. The absolute number of PC^+^-P (PC^−^-P) and *Psm*^+^ (*Psm*^−^) genes within a particular category is indicated on the respective horizontal bar. Asterisks next to the bars indicate significant enrichment (or depletion) of gene categories in PC^+^-P (PC^−^-P) or *Psm*^+^ (*Psm*^−^) genes (**p < 0.001; *p < 0.05; Fisher's exact test).

When the plants were treated with both the bacterial pathogen and the compound C234, the functional categories of ‘signalling’, ‘transport’ and ‘hormone metabolism’ were significantly enriched among the PC^+^-P genes (figure 2*e*); on the other hand, among PC^−^-P genes the significantly enriched categories were ‘photosynthesis’, ‘tetrapyrrole synthesis’, ‘DNA’ and ‘RNA’ (figure 2*f*). To acquire a better representation of the C234 effect on plant response to the pathogen, we analysed the MapMan bins enriched in the 1654 PC^+^-P genes and the 1822 PC^−^-P genes form the *Psm*^+^ and *Psm*^−^ genes, respectively, i.e. the responsive genes extended from *Psm*-infection owing to C234 application. We found that the categories of ‘nucleotides transport’, ‘vesicle transport’, ‘MAP kinases’, ‘G-proteins’, ‘glycosylation’ and ‘vacuolar-sorting protein SNF7’ showed significant enrichments among the PC^+^-P genes (figure 3*a*; electronic supplementary material, figure S4), whereas the ‘protein assembly and cofactor ligation’, ‘chromatin structure. histone’, ‘deoxynucleotide metabolism’ and ‘light reaction’ categories were significantly enriched among the PC-P^−^ genes compared to the *Psm*^−^ genes (figure 3*b*). These results suggested that the compound C234 disturbed multiple physiological processes, including metabolism, photosynthesis, transport and protein complex assembly, which, as a result, exacerbated the plant disease development.

**Figure 4.**
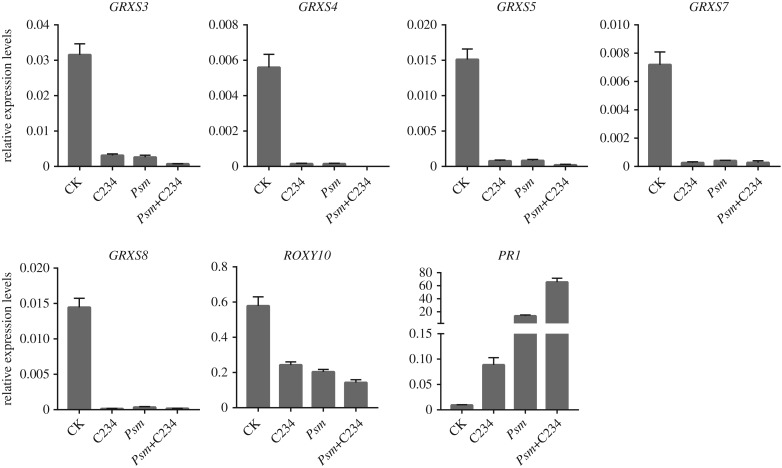
Effects of C234 and *Psm* on the expression of six glutaredoxin genes in *Arabidopsis* leaves, as measured by qRT-PCR. Data are shown as means ± s.d. (*n* = 3). The glutaredoxin genes were genes downregulated by the treatments. *PR1* (AT2G14610) was included in the analysis as a reference of upregulation.

Glutaredoxins are anti-oxidant enzymes and involved in reactive oxygen species scavenging [27]. Notably, the C234 treatment repressed the expression of a cluster of glutaredoxin genes, including *GRXS3* (*AT4G15700*), *GRXS4* (*AT4G15680*), *GRXS5* (*AT4G15690*), *GRXS7* (*AT4G15670*) and *GRXS8* (*AT4G15660*) which, together with *ROXY10 (AT5G18600)*, encode the CC-type glutaredoxin family proteins and were significantly enriched in the C234^−^ genes (electronic supplementary material, table S3). Further analysis by qRT-PCR confirmed the downregulation of the six glutaredoxin genes in the *Psm-* and (*Psm*+C234)-treated samples (figure 4). Moreover, the mean-fold transcriptional change (0.18, C234/CK) of the five clustered glutaredoxin genes (*AtGRXS3/4/5/7/8*) was considerably lower than that (0.54) of the remaining genes, thus they were among the most strongly downregulated genes in the C234-treated samples. This result is consistent with the previous finding that At3g62950, a glutaredoxin-like protein was downregulated in *vitamin e2* (*vte2*) mutant, in which there was a massive increase in the levels of the nonenzymatic lipid peroxidation products [26]. It has been reported that *AtGRXS3/4/5/7/8* are negative regulators of plant primary root growth in response to nitrate [28,29]. However, expression of glutaredoxin genes in *Arabidopsis* could be induced by malondialdehyde, an RES produced by non-enzymatic lipid oxidation reactions [14]. Together, these data suggest that the RESs may affect the glutaredoxin genes with different yet unidentified mechanisms; alternatively, the changed glutaredoxin levels are merely a result of the distorted cellular redox status induced by the RES.

## 4. Discussion

In the current study, the transcriptome analysis compared the genes differentially expressed in response to C234, *Psm* and (*Psm*+C234). The genes induced by all three treatments are mainly involved in responses to biotic/abiotic stresses. Interestingly, the (*Psm*+C234) treatment affected many more genes than the *Psm*-inoculation alone. Investigation into the PC^+^/^−^-P genes demonstrated that, in addition to the defence responses elicited by *Psm*, application of C234 during *Psm* infection led to an over-response of the plant, bringing disturbance in various physiological processes, including particularly, protein transport and protein complex assembly (figure 2*e*,*f*). These abnormalities revealed by the extended and dramatic transcriptional changes may account for the increased susceptibility to pathogen infection and faster symptom development. C234, bearing an α,β-unsaturated keto group, is a member of the RES compounds. Although many RESs are non-enzymatic fatty acid oxygenation products [26], some are derived from enzymatic catalysis. The RES structure is widely encountered in phytohormone compounds or secondary metabolites, such as abscisic acid, oxophytodienoic acid, 5-deoxystrigol, tomatid-4-en-3-one (an intermediate of tomatidine biosynthesis) [30] and strictosidine (a central monoterpene indole alkaloid intermediate) [31], in addition to lipid peroxidation products [26]. Investigations of the enzymatic RES, especially the biosynthetic intermediates, are of special interests as they are usually hidden or in low content in plant tissues. Accumulations of these compounds arose commonly during investigation when the respective biosynthetic pathways were interrupted genetically or biochemically, which may provide fresh insight into the regulatory mechanism of plant secondary metabolism and help find new active natural products. For example, in *Catharanthus roseus* the nitrate/peptide family (NPF) transporter CrNPF2.9 plays a key role in the monoterpene indole alkaloid biosynthesis by exporting the cytotoxic intermediate strictosidine from the vacuole; silencing of *CrNPF2.9* induced the strictosidine accumulation and subsequently caused the extensive tissue death [31].

In cotton plants the gossypol pathway intermediate C234 was undetectable both before and after *R. solani* inoculation, although at the same time other intermediates were induced to accumulate by the pathogen (figure 1*b*,*c*). This is consistent with the fact that the P450 monooxygenase CYP71BE79 is catalytically highly efficient in transforming C234 into 8,11-dihydroxy-7-keto-δ-cadinene, and its maximum activity is more than ten times higher than that of the other identified enzymes of the gossypol pathway [13]. Although other gossypol pathway intermediates may also contain the α,β-unsaturated carbonyl, to date only C234 is found to have the activity in enhancing pathogen susceptibility, probably owing to its specific structure. The toxicity of C234 may have exerted a selection pressure on the regulation of gossypol biosynthesis, and cotton plants have evolved a highly active P450 enzyme to prevent the accumulation of the cytotoxic intermediate.

The working mechanism of electrophile perception of RES has been partly elucidated in mammals, in which the Kelch-like ECH-associated protein 1 (Keap1) and nuclear factor-erythroid 2-related factor 2 (Nrf2) play a critical role [32]. A more recent report demonstrated that itaconate, which contains an electrophilic α,β-unsaturated carboxylic acid, directly alkylates the protein Keap1, enabling Nrf2 to promote downstream gene expressions [33]. However, the Nrf2 homologues have not been found in plants and whether a similar signalling pathway exists in plants remains an open question. At present we cannot distinguish between direct (e.g. specific binding to a receptor) and the indirect (e.g. perturbation of membranes by oxidative stress) effects. Nevertheless, comprehensive analysis of the global changes in gene expressions induced by C234 during pathogen infection should help identify factors that contribute to plant defence and shed new light on the evolution of the biosynthetic pathway of specialized metabolites in plants.

## Supplementary Material

Supplementary tables and figures

## Data accessibility

Additional data are provided as the electronic supplementary material.

## References

[1] Dixon RA 2001 Natural products and plant disease resistance. Nature 411, 843–847. (10.1038/35081178) 11459067

[2] Smith CJ 1996 Accumulation of phytoalexins: defence mechanism and stimulus response system. New Phytol. 132, 1–45. (10.1111/j.1469-8137.1996.tb04506.x) 33863060

[3] Wink M 2003 Evolution of secondary metabolites from an ecological and molecular phylogenetic perspective. Phytochemistry 64, 3–19. (10.1016/S0031-9422(03)00300-5) 12946402

[4] Kemen AC, Honkanen S, Melton RE, Findlay KC, Mugford ST, Hayashi K, Haralampidis K, Rosser SJ, Osbourn A 2014 Investigation of triterpene synthesis and regulation in oats reveals a role for beta-amyrin in determining root epidermal cell patterning. Proc. Natl Acad. Sci. USA 111, 8679–8684. (10.1073/pnas.1401553111) 24912185 PMC4060722

[5] Nützmann HW, Huang A, Osbourn A 2016 Plant metabolic clusters—from genetics to genomics. New Phytol. 211, 771–789. (10.1111/nph.13981) 27112429 PMC5449196

[6] Bell AA, Stipanovic RD, O'Brien DH, Fryxell PA 1978 Sesquiterpenoid aldehyde quinones and derivatives in pigment glands of *Gossypium*. Phytochemistry 17, 1297–1305. (10.1016/S0031-9422(00)94578-3)

[7] Chappell J, Nable R 1987 Induction of sesquiterpenoid biosynthesis in tobacco cell suspension cultures by fungal elicitor. Plant Physiol. 85, 469–473. (10.1104/pp.85.2.469) 16665722 PMC1054280

[8] Huffaker A, Kaplan F, Vaughan MM, Dafoe NJ, Ni X, Rocca JR, Alborn HT, Teal PE, Schmelz EA 2011 Novel acidic sesquiterpenoids constitute a dominant class of pathogen-induced phytoalexins in maize. Plant Physiol. 156, 2082–2097. (10.1104/pp.111.179457) 21690302 PMC3149930

[9] Alford BB, Liepa GU, Vanbeber AD 1996 Cottonseed protein: what does the future hold? Plant Foods Hum. Nutr. 49, 1 (10.1007/BF01092517) 9139299

[10] Sunilkumar G, Campbell LM, Puckhaber L, Stipanovic RD, Rathore KS 2006 Engineering cottonseed for use in human nutrition by tissue-specific reduction of toxic gossypol. Proc. Natl Acad. Sci. USA 103, 18 054–18 059. (10.1073/pnas.0605389103) PMC1838705 17110445

[11] Chen XY, Chen Y, Heinstein P, Davisson VJ 1995 Cloning, expression, and characterization of (+)-δ-cadinene synthase: a catalyst for cotton phytoalexin biosynthesis. Arch. Biochem. Biophys. 324, 255–266. (10.1006/abbi.1995.0038) 8554317

[12] Chen XY, Wang M, Chen Y, Davisson VJ, Heinstein P 1996 Cloning and heterologous expression of a second (+)-δ-cadinene synthase from *Gossypium arboreum*. J. Nat. Prod. 59, 944–951. (10.1021/np960344w) 8904844

[13] Tian X et al. 2018 Characterization of gossypol biosynthetic pathway. Proc. Natl Acad. Sci. USA 115, E5410–E5418. (10.1073/pnas.1805085115) 29784821 PMC6003316

[14] Farmer EE, Davoine C 2007 Reactive electrophile species. Curr. Opin. Plant Biol. 10, 380–386. (10.1016/j.pbi.2007.04.019) 17646124

[15] Guo YH, Yu YP, Wang D, Wu CA, Yang GD, Huang JG, Zheng CC 2009 GhZFP1, a novel CCCH-type zinc finger protein from cotton, enhances salt stress tolerance and fungal disease resistance in transgenic tobacco by interacting with GZIRD21A and GZIPR5. New Phytol. 183, 62–75. (10.1111/j.1469-8137.2009.02838.x) 19402879

[16] Clarke JD, Liu Y, Klessig DF, Dong X 1998 Uncoupling PR gene expression from NPR1 and bacterial resistance: characterization of the dominant *Arabidopsis* *cpr6-1* mutant. Plant Cell 10, 557–569. (10.1105/tpc.10.4.557) 9548982 PMC144011

[17] Anders S, Huber W 2010 Differential expression analysis for sequence count data. Genome Biol. 11, R106 (10.1186/gb-2010-11-10-r106) 20979621 PMC3218662

[18] Hartmann M et al. 2018 Flavin monooxygenase-generated N-hydroxypipecolic acid is a critical element of plant systemic immunity. Cell 173, 1–14. (10.1016/j.cell.2018.02.049) 29576453

[19] Wang JY, Cai Y, Gou JY, Mao YB, Xu YH, Jiang WH, Chen XY 2004 VdNEP, an elicitor from *Verticillium dahliae*, induces cotton plant wilting. Appl. Environ. Microbiol. 70, 4989–4995. (10.1128/AEM.70.8.4989-4995.2004) 15294839 PMC492334

[20] Lee S, Badieyan S, Bevan DR, Herde M, Gatz C, Tholl D 2010 Herbivore-induced and floral homoterpene volatiles are biosynthesized by a single P450 enzyme (CYP82G1) in *Arabidopsis*. Proc. Natl Acad. Sci. USA 107, 21 205–21 210. (10.1073/pnas.1009975107) PMC3000306 21088219

[21] Herde M, Gartner K, Kollner TG, Fode B, Boland W, Gershenzon J, Gatz C, Tholl D 2008 Identification and regulation of TPS04/GES, an *Arabidopsis* geranyllinalool synthase catalyzing the first step in the formation of the insect-induced volatile C16-homoterpene TMTT. Plant Cell 20, 1152–1168. (10.1105/tpc.106.049478) 18398052 PMC2390743

[22] Schuhegger R, Nafisi M, Mansourova M, Petersen BL, Olsen CE, Svatos A, Halkier BA, Glawischnig E 2006 CYP71B15 (PAD3) catalyzes the final step in camalexin biosynthesis. Plant Physiol. 141, 1248–1254. (10.1104/pp.106.082024) 16766671 PMC1533948

[23] Zhou N, Tootle TL, Glazebrook J 1999 *Arabidopsis* PAD3, a gene required for camalexin biosynthesis, encodes a putative cytochrome P450 monooxygenase. Plant Cell 11, 2419–2428. (10.1105/tpc.11.12.2419) 10590168 PMC144139

[24] van Loon LC, Rep M, Pieterse CM 2006 Significance of inducible defense-related proteins in infected plants. Annu. Rev. Phytopathol. 44, 135–162. (10.1146/annurev.phyto.44.070505.143425) 16602946

[25] Almeras E, Stolz S, Vollenweider S, Reymond P, Mene-Saffrane L, Farmer EE 2003 Reactive electrophile species activate defense gene expression in *Arabidopsis*. Plant J. 34, 205–216. (10.1046/j.1365-313X.2003.01718.x) 12694595

[26] Sattler SE, Mene-Saffrane L, Farmer EE, Krischke M, Mueller MJ, DellaPenna D 2006 Nonenzymatic lipid peroxidation reprograms gene expression and activates defense markers in *Arabidopsis* tocopherol-deficient mutants. Plant Cell 18, 3706–3720. (10.1105/tpc.106.044065) 17194769 PMC1785394

[27] Rouhier N, Lemaire SD, Jacquot JP 2008 The role of glutathione in photosynthetic organisms: emerging functions for glutaredoxins and glutathionylation. Annu. Rev. Plant Biol. 59, 143–166. (10.1146/annurev.arplant.59.032607.092811) 18444899

[28] Patterson K, Walters LA, Cooper AM, Olvera JG, Rosas MA, Rasmusson AG, Escobar MA 2016 Nitrate-regulated glutaredoxins control *Arabidopsis* primary root growth. Plant Physiol. 170, 989–999. (10.1104/pp.15.01776) 26662603 PMC4734588

[29] Walters LA, Escobar MA 2016 The *AtGRXS3/4/5/7/8* glutaredoxin gene cluster on *Arabidopsis thaliana* chromosome 4 is coordinately regulated by nitrate and appears to control primary root growth. Plant Signal. Behav. 11, e1171450 (10.1080/15592324.2016.1171450) 27049601 PMC4883855

[30] Sonawane PD et al. 2018 Short-chain dehydrogenase/reductase governs steroidal specialized metabolites structural diversity and toxicity in the genus *Solanum*. Proc. Natl Acad. Sci. USA 115, E5419–E5428. (10.1073/pnas.1804835115) 29784829 PMC6003347

[31] Payne RM et al. 2017 An NPF transporter exports a central monoterpene indole alkaloid intermediate from the vacuole. Nat. Plants 3, 16208 (10.1038/nplants.2016.208) 28085153 PMC5238941

[32] Satoh T, Okamoto SI, Cui J, Watanabe Y, Furuta K, Suzuki M, Tohyama K, Lipton SA 2006 Activation of the Keap1/Nrf2 pathway for neuroprotection by electrophilic [correction of electrophillic] phase II inducers. Proc. Natl Acad. Sci. USA 103, 768–773. (10.1073/pnas.0505723102) 16407140 PMC1334635

[33] Mills EL et al. 2018 Itaconate is an anti-inflammatory metabolite that activates Nrf2 via alkylation of KEAP1. Nature 556, 113–117. (10.1038/nature25986) 29590092 PMC6047741

