# Supplementary material for: A gossypol biosynthetic intermediate disturbs plant defence response
Source: Philos Trans R Soc Lond B Biol Sci. 2019 Jan 14;374(1767):20180319. doi: 10.1098/rstb.2018.0319 (PMC6367145; doi:10.1098/rstb.2018.0319)
Supplement: Supplementary tables and figures [file rstb20180319supp1.docx]

*Author for correspondence (xychen@sibs.ac.cn).

†Present address:

A gossypol biosynthetic intermediate disturbs plant defense response

Xiu Tian ^1,2^, Xin Fang ^2^, Jin-Quan Huang ^2^, Ling-Jian Wang ^2^, Ying-Bo Mao ^3^, Xiao-Ya Chen ^2^**^*^**

^1^ School of Life Sciences, Nanjing University, Nanjing 210023, China.

^2^ National Key Laboratory of Plant Molecular Genetics, ^3^ CAS Key Laboratory of Insect Developmental and Evolutionary Biology, CAS Center for Excellence in Molecular Plant Sciences, Shanghai Institute of Plant Physiology and Ecology, Chinese Academy of Sciences, Shanghai 200032, China

**Author for correspondence (xychen@sibs.ac.cn)*

**Electronic supplementary material**

**Contents**

1. Supplementary Figures

2. Supplementary Tables

**1. Supplementary Figures**

**Figure S1.** The 8-hydroxy-7-keto-δ-cadinene (C234) content in the VIGS-*CYP71BE79* cotton plants. The content was determined by HPLC at 254nm. Plant tissues were harested two weeks post the VIGS treatment on the 10-day old cotton seedlings and data were derived from six biological replicates, and the mean ± SD are shown.

**
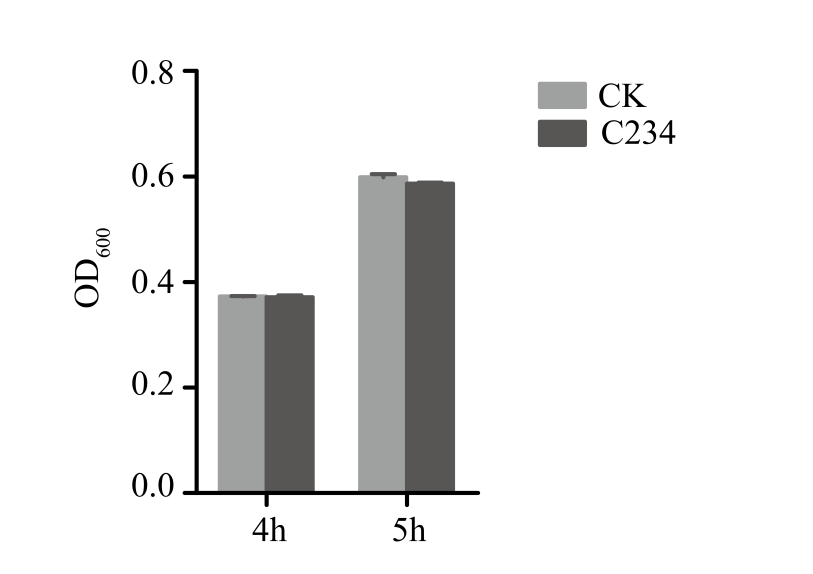
**

**Figure S2.** The compound 8-hydroxy-7-keto-δ-cadinene (C234, at 200 μM) did not affect the phytopathogenic bacterium (*Pseudomonas syringae* pv. *maculicola* (*Psm*) ES4326) growth in culture condition. Data were derived from six biological replicates, and the mean ± SD are shown.

**
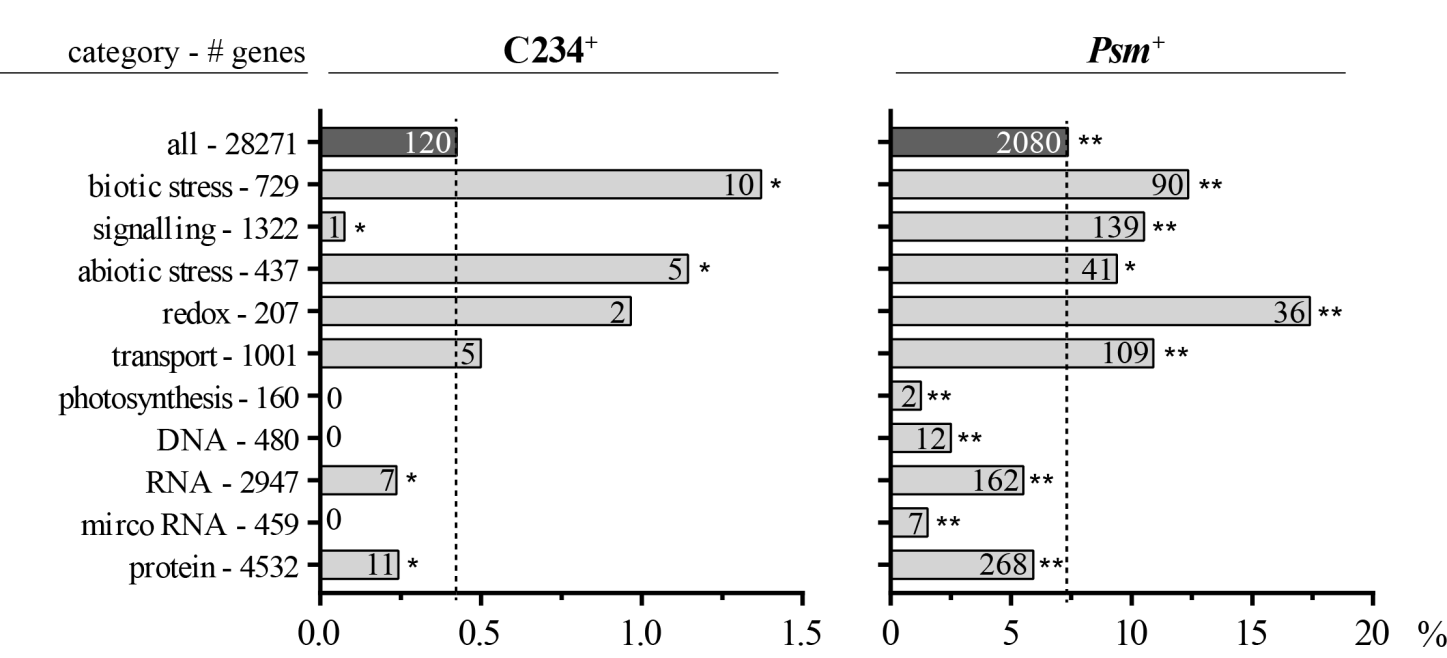
**

**Figure S3.** Proportions of C234^+^ (left) and *Psm*^+^ (right) genes in main MapMan functional categories. The total number (#) of genes in each category is indicated on the left. The absolute number of C234^+^ and *Psm*^+^ genes within a particular gene category is indicated on the horizontal bars. Asterisks indicate significant enrichment (or depletion) of gene categories in C234^+^ or *Psm*^+^ genes (**p < 0.001; *p < 0.05; Fisher’s exact test).

**
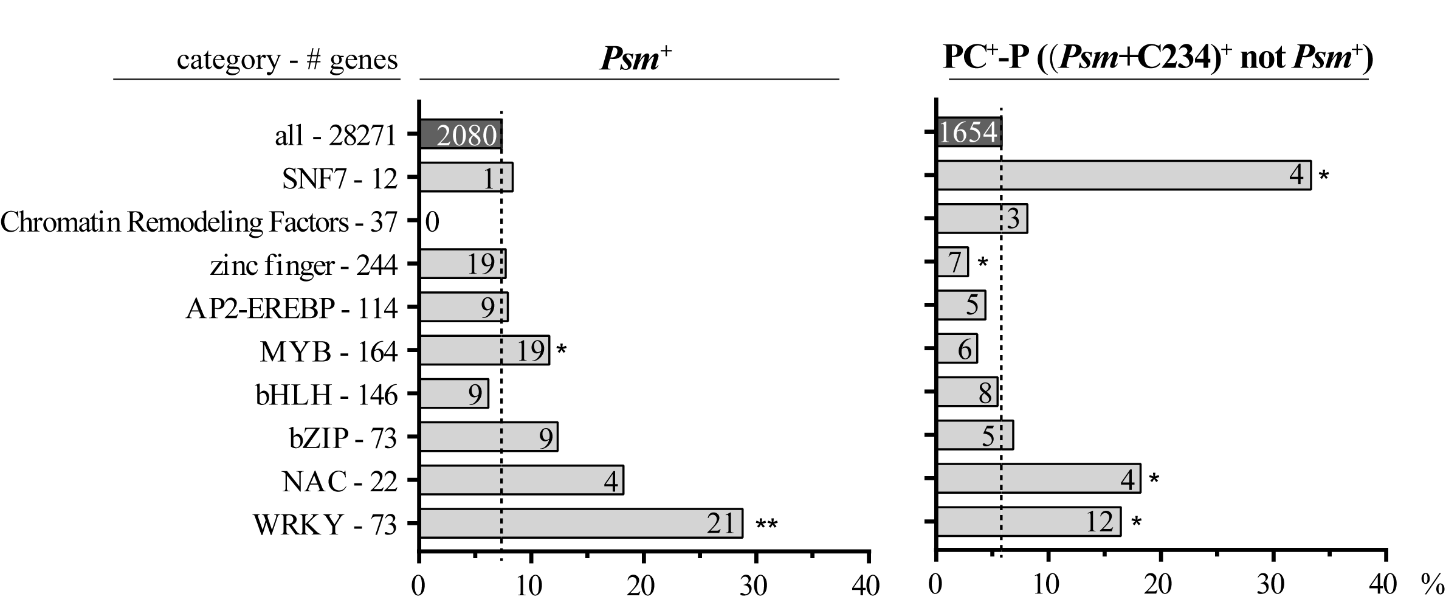
**

**Figure S4.** Proportions of *Psm*^+^ and PC^+^-P genes in defined gene groups representing MapMan functional categories and *Arabidopsis* main transcription factor families. Asterisks indicate significant enrichment (or depletion) of gene categories in *Psm*^+^ or PC^+^-P genes (**p < 0.001; *p < 0.05; Fisher’s exact test).

**1. Supplementary Tables**

**Table S1.** Primers used in this investigation.

| **Primer name** | **Primer sequence (5'-3')** | **Target gene** |
| --- | --- | --- |
| GRXS3-qrtF | GCAAGACAGTTTTCAAATATCTATCAC | AT4G15700 |
| GRXS3-qrtR | CAAGCTGCCCTCCTATGAAC | AT4G15700 |
| GRXS4-qrtF | CGCTCTTCGAACACATTTCA | AT4G15680 |
| GRXS4-qrtR | GCCCTCCAATAAACACCACA | AT4G15680 |
| GRXS5-qrtF | AACCGCTCCCTTGTTCCTAT | AT4G15690 |
| GRXS5-qrtR | TTCTTGTGTATGTTTTCTTTGTTCA | AT4G15690 |
| GRXS7-qrtF | TAGACCTTGGCGTAAATCC | AT4G15670 |
| GRXS7-qrtR | TGCCCTCCTATGAACACC | AT4G15670 |
| GRXS8-qrtF | TGCATGTCACACACAATCAAG | AT4G15660 |
| GRXS8-qrtR | AAAGCCATAAAGCCCCAAA | AT4G15660 |
| ROXY10-qrtF | CAAATCCAGCGGTTTACGA | AT5G18600 |
| ROXY10-qrtR | TGGCTCCACCGACCAACTC | AT5G18600 |
| PR1-qrtF | CTCGAAAGCTCAAGATAGCCC | AT2G14610 |
| PR1-qrtR | TTCCACCATTGTTACACCTCAC | AT2G14610 |
| UBQ5-qrtF | TTGAATCATCCGACACCATC | AT3G62250 |
| UBQ5-qrtR | GCTCCACAGGTTGCGTTAG | AT3G62250 |

**Table S2.** Comparison of the fold of change of the C234^+^, *Psm*^+^ and (*Psm+*C234)^+^ genes. Numbers show log2-fold change in expression in C234-treated, *Psm*-inoculated and *Psm*C234-inoculated samples referred to the CK samples. The genes listed are responsive to all the three treatments.

| gene_name | C234/CK | *Psm*/CK | *Psm*C234/CK | (*Psm+*C234)/*Psm* | FDR | Description |
| --- | --- | --- | --- | --- | --- | --- |
| AT2G37760 | 1.26 | 1.71 | 2.94 | 1.23 | <0.05 | AKR4C8 |
| AT5G24200 | 1.39 | 4.64 | 5.48 | 0.84 | <0.05 | alpha/beta-Hydrolases superfamily protein; |
| AT4G12490 | 1.73 | 1.46 | 3.97 | 2.50 | <0.05 | AZI3 |
| AT1G05710 | 1.48 | 1.95 | 2.88 | 0.93 | <0.05 | basic helix-loop-helix (bHLH) DNA-binding superfamily protein |
| AT5G20230 | 1.24 | 3.56 | 4.90 | 1.34 | <0.05 | BCB |
| AT3G50930 | 0.87 | 2.99 | 3.50 | 0.51 | <0.05 | BCS1 |
| AT3G57260 | 1.90 | 5.82 | 6.27 | 0.45 | <0.05 | beta 1,3-glucanase |
| AT3G13380 | 1.10 | 1.88 | 2.71 | 0.82 | <0.05 | BRL3 |
| AT2G41410 | 0.68 | 1.75 | 2.17 | 0.42 | <0.05 | Calcium-binding EF-hand family protein |
| AT2G46600 | 0.63 | 1.31 | 1.57 | 0.26 | <0.05 | Calcium-binding EF-hand family protein |
| AT1G19020 | 1.52 | 2.15 | 3.41 | 1.26 | <0.05 | CDP-diacylglycerol-glycerol-3-phosphate 3-phosphatidyltransferase; |
| AT2G43570 | 2.02 | 5.56 | 6.84 | 1.27 | <0.05 | CHI |
| AT2G17120 | 1.13 | 1.31 | 1.91 | 0.60 | <0.05 | CL-1 |
| AT5G27420 | 1.43 | 2.68 | 3.97 | 1.29 | <0.05 | CNI1 |
| AT5G60950 | 1.26 | 2.81 | 2.65 | -0.16 | <0.05 | COBL5 |
| AT4G04490 | 1.37 | 3.53 | 4.70 | 1.17 | <0.05 | CRK36 |
| AT4G11890 | 1.24 | 2.98 | 4.22 | 1.23 | <0.05 | CRK45 |
| AT3G26210 | 0.88 | 2.71 | 4.04 | 1.33 | <0.05 | CYP71B23 |
| AT3G14620 | 0.69 | 1.46 | 2.46 | 1.00 | <0.05 | CYP72A8 |
| AT3G25180 | 4.94 | 2.84 | 5.65 | 2.80 | <0.05 | CYP82G1 |
| AT3G49340 | 5.15 | 3.70 | 4.63 | 0.93 | <0.05 | Cysteine proteinases superfamily protein |
| AT4G23210 | 1.08 | 2.97 | 3.71 | 0.73 | <0.05 | cysteine-rich RLK (RECEPTOR-like protein kinase) 13; |
| AT4G10500 | 1.48 | 5.50 | 7.03 | 1.53 | <0.05 | DLO1 |
| AT1G03820 | 3.53 | 0.96 | 3.53 | 2.57 | <0.05 | E6-like protein |
| AT4G37990 | 4.47 | 2.45 | 6.29 | 3.83 | <0.05 | ELI3 |
| AT3G27400 | 4.30 | 0.59 | 4.17 | 3.57 | <0.05 | Encodes a pectate lyase involved in response to nematodes. |
| AT1G29330 | 0.97 | 2.11 | 2.67 | 0.56 | <0.05 | ER lumen protein retaining receptor family protein |
| AT5G09440 | 1.32 | 2.03 | 3.31 | 1.28 | <0.05 | EXL4 |
| AT1G79530 | 1.19 | 0.67 | 1.35 | 0.68 | <0.05 | GAPCP-1 |
| AT5G55050 | 0.82 | 1.23 | 1.44 | 0.21 | <0.05 | GDSL-motif esterase/acyltransferase/lipase |
| AT1G54010 | 3.46 | 7.58 | 6.28 | -1.30 | <0.05 | GLL23 |
| AT1G02920 | 1.43 | 3.49 | 4.09 | 0.60 | <0.05 | GST11 |
| AT1G02930 | 2.32 | 3.91 | 4.69 | 0.78 | <0.05 | GSTF6 |
| AT2G46680 | 1.00 | 2.00 | 2.57 | 0.57 | <0.05 | HB-7 |
| AT5G42020 | 0.75 | 1.11 | 1.75 | 0.64 | <0.05 | Heat shock protein 70 (Hsp 70) family protein |
| AT3G50480 | 1.47 | 3.58 | 4.14 | 0.55 | <0.05 | HR4 |
| AT5G49280 | 1.04 | 1.50 | 1.86 | 0.36 | <0.05 | hydroxyproline-rich glycoprotein family protein |
| AT4G18630 | 3.21 | 1.68 | 3.97 | 2.29 | <0.05 | hypothetical protein (DUF688); |
| AT3G14060 | 2.25 | 3.04 | 4.89 | 1.85 | <0.05 | hypothetical protein; |
| AT1G21100 | 3.02 | 3.39 | 4.99 | 1.60 | <0.05 | IGMT1 |
| AT5G23020 | 0.85 | 1.22 | 1.98 | 0.75 | <0.05 | IMS2 |
| AT5G12930 | 1.12 | 1.86 | 2.68 | 0.82 | <0.05 | inactive rhomboid protein; |
| AT2G39330 | 1.11 | 4.04 | 4.22 | 0.17 | <0.05 | jacalin-related lectin 23; |
| AT2G28500 | 2.62 | 1.26 | 3.46 | 2.20 | <0.05 | LBD11 |
| AT1G19610 | 2.09 | 1.54 | 2.94 | 1.40 | <0.05 | LCR78 |
| AT3G22600 | 1.82 | 3.67 | 5.35 | 1.67 | <0.05 | LTPG5 |
| AT3G23560 | 0.89 | 1.59 | 2.35 | 0.76 | <0.05 | MATE efflux family protein |
| AT3G45290 | 0.87 | 1.47 | 2.27 | 0.79 | <0.05 | MLO3 |
| AT2G47800 | 0.81 | 2.09 | 2.69 | 0.60 | <0.05 | MRP4 |
| AT5G09590 | 0.60 | 0.69 | 1.13 | 0.44 | <0.05 | MTHSC70-2 |
| AT3G29250 | 2.31 | 3.47 | 5.24 | 1.77 | <0.05 | NAD(P)-binding Rossmann-fold superfamily protein |
| AT3G22550 | 0.86 | 1.34 | 1.42 | 0.08 | <0.05 | NAD(P)H-quinone oxidoreductase subunit, putative (DUF581) |
| AT1G60730 | 0.98 | 2.32 | 3.50 | 1.19 | <0.05 | NAD(P)-linked oxidoreductase superfamily protein |
| AT4G05020 | 0.87 | 1.83 | 2.37 | 0.54 | <0.05 | NDB2 |
| AT3G50910 | 0.69 | 1.41 | 1.78 | 0.37 | <0.05 | netrin receptor DCC |
| AT5G42050 | 0.77 | 1.73 | 2.86 | 1.13 | <0.05 | NRP |
| AT4G29520 | 1.00 | 1.66 | 1.91 | 0.25 | <0.05 | Nucleophosmin |
| AT1G76930 | 1.73 | 1.79 | 2.81 | 1.02 | <0.05 | ORG5 |
| AT4G36648 | 0.64 | 2.37 | 3.22 | 0.85 | <0.05 | other_RNA |
| AT1G04980 | 1.88 | 3.25 | 3.48 | 0.23 | <0.05 | PDI10 |
| AT1G21750 | 0.59 | 1.20 | 1.65 | 0.45 | <0.05 | PDIL1-1 |
| AT1G17745 | 0.81 | 2.39 | 3.23 | 0.84 | <0.05 | PGDH |
| AT1G43910 | 1.24 | 3.37 | 3.88 | 0.50 | <0.05 | P-loop containing nucleoside triphosphate hydrolases superfamily protein |
| AT4G02330 | 3.11 | 0.74 | 3.31 | 2.56 | <0.05 | PME41 |
| AT2G18660 | 1.67 | 5.78 | 6.73 | 0.95 | <0.05 | PNP-A |
| AT4G23680 | 3.13 | 3.36 | 6.75 | 3.39 | <0.05 | Polyketide cyclase/dehydrase and lipid transport superfamily protein |
| AT2G14610 | 2.39 | 7.55 | 8.81 | 1.26 | <0.05 | PR1 |
| AT1G75040 | 1.87 | 3.88 | 4.48 | 0.61 | <0.05 | PR5 |
| AT1G48210 | 0.93 | 2.24 | 3.37 | 1.12 | <0.05 | Protein kinase superfamily protein |
| AT3G49120 | 0.98 | 1.97 | 3.70 | 1.73 | <0.05 | PRX34 |
| AT3G25010 | 0.79 | 3.45 | 4.61 | 1.16 | <0.05 | receptor like protein 41 |
| AT5G27060 | 3.50 | 4.62 | 6.91 | 2.29 | <0.05 | receptor like protein 53 |
| AT1G63840 | 0.88 | 1.40 | 3.46 | 2.06 | <0.05 | RING/U-box superfamily protein |
| AT5G10380 | 0.85 | 3.10 | 3.61 | 0.51 | <0.05 | RING1 |
| AT2G32680 | 0.99 | 3.06 | 3.79 | 0.72 | <0.05 | RLP23 |
| AT2G29350 | 2.47 | 4.88 | 6.99 | 2.11 | <0.05 | SAG13 |
| AT2G41800 | 4.58 | 0.95 | 4.93 | 3.97 | <0.05 | TEB |
| AT5G38900 | 4.14 | 5.37 | 6.94 | 1.57 | <0.05 | Thioredoxin superfamily protein; |
| AT5G44572 | 1.53 | 1.81 | 2.91 | 1.10 | <0.05 | transmembrane protein |
| AT2G18690 | 1.21 | 3.89 | 5.24 | 1.35 | <0.05 | transmembrane protein |
| AT5G44570 | 2.61 | 4.26 | 5.42 | 1.15 | <0.05 | transmembrane protein |
| AT5G08240 | 1.55 | 4.12 | 5.05 | 0.93 | <0.05 | transmembrane protein; |
| AT5G46350 | 2.55 | 2.29 | 4.12 | 1.82 | <0.05 | WRKY8 |
| AT5G17000 | 0.73 | 0.63 | 1.55 | 0.92 | <0.05 | Zinc-binding dehydrogenase family protein |

**Table S3.** GO-term enrichments of C234-downregulated (C234^-^) genes.

| GO term | Ontology | Description | Number in input list | Number in BG/Ref | FDR |
| --- | --- | --- | --- | --- | --- |
| GO:0045454 | P | cell redox homeostasis | 8 * | 136 | 3.80E-07 |
| GO:0019725 | P | cellular homeostasis | 8 * | 328 | 0.00014 |
| GO:0008794 | F | arsenate reductase (glutaredoxin) activity | 6 * | 14 | 1.20E-10 |
| GO:0030611 | F | arsenate reductase activity | 6 * | 15 | 1.30E-10 |
| GO:0015035 | F | protein disulfide oxidoreductase activity | 8 * | 94 | 1.40E-09 |
| GO:0015036 | F | disulfide oxidoreductase activity | 8 * | 102 | 2.20E-09 |
| GO:0016667 | F | oxidoreductase activity, acting on sulfur group of donors | 8 * | 283 | 3.90E-06 |
| GO:0009055 | F | electron carrier activity | 9 * | 525 | 3.40E-05 |

*All the six glutaredoxin genes, *AtGRXS3/4/5/7/8* and *ROXY10* are included. P, biological process; F, molecular function.
